# Bibliometric analysis of surface electromyography trends in stroke rehabilitation research

**DOI:** 10.3389/fneur.2025.1568797

**Published:** 2025-08-11

**Authors:** Zhiping Liao, Jianhua Li, Fangchao Wu, Yunxiang Xia, Yangzheng Li, Lina Ma, Lei Wu

**Affiliations:** ^1^Department of Rehabilitation Medicine, Sir Run Run Shaw Hospital, Zhejiang University School of Medicine, Hangzhou, China; ^2^Department of General Medicine, Hangzhou Third People’s Hospital, Hangzhou, China; ^3^Department of Acupuncture and Moxibustion, The Third Affiliated Hospital of Zhejiang Chinese Medical University, Hangzhou, China

**Keywords:** stroke, sEMG, bibliometric analysis, rehabilitation, clinical application

## Abstract

Stroke, as a common clinical disease, has seen its mortality rate rising globally. Muscle dysfunction after stroke seriously affects the limb function of patients. Surface electromyography (sEMG), often referred to as the ECG of muscles, can effectively evaluate changes in muscle function in stroke patients. In this study, we searched for articles in the Web of Science database up to December 2023 and utilized the “bibliometrix” package in R software (version 4.3.3) to analyze bibliographic information. We identified 908 articles published between 1979 and 2023. Citation analysis revealed 18 articles with over 100 citations. Our bibliometric analysis included 908 articles from 1,241 institutions across 49 countries or regions, with a gradual increase in the number of articles over time. The United States led in the number of publications, followed by China, South Korea, and Japan. The Northwestern University and Shirley Ryan AbilityLab Joint Research Consortium (NU-SRAL) published the most articles with 151, accounting for 16.6% of the total. Sun Yat-sen University followed with 49 articles (5.3%), and the University of British Columbia with 35 articles (3.6%). Zhang X was the most prolific author, publishing 25 articles, while Lay B. S. was the most influential, with 120 citations. The articles were published in 371 journals, with the Journal of Electromyography and Kinesiology having the highest number, totaling 37 articles, which is about 10% of the total. The most frequent keywords were “stroke” and “rehabilitation.” Our analysis indicates a significant rise in sEMG research on stroke since 2009, suggesting that this field is a promising area for future study.

## 1 Introduction

Stroke is a leading cause of death and disability. According to a recent study in 2016 (1), the global risk of stroke in people aged 25 years or older was about 24.9%, which was an increase from 22.8% in 1990. The risk was found to be similar across both men and women (2). With an estimated 101 million stroke patients living worldwide in 2019 (3), approximately 34% of total global healthcare expenditures are also spent on stroke. In the United States, the average per person healthcare cost of stroke is estimated at USD 140,048 (4). Approximately 50 to 70 percent of stroke patients with long-term complications experience motor dysfunction (5, 6). Motor dysfunction (such as muscle weakness and abnormal posture control) significantly impairs patients’ daily living capabilities, participation levels, and social engagement enthusiasm. The decline in muscle function in post-stroke motor dysfunction manifests as excessive muscle co-contraction, diminished muscle strength, and dystonia development. Consequently, the assessment and management of muscle function are critical strategies for ameliorating movement disorders in stroke patients (7).

Surface electromyography (sEMG), a technique that measures muscle electrical activity through electrodes on the skin surface, serves as an appropriate tool for evaluating muscle function and discerning between physiological and pathological states (8, 9). In reality, sEMG has been utilized in neurological rehabilitation to evaluate muscle function since 1979, encompassing assessments of muscle activation (10) and co-contraction (11), along with dystonia (12). The clinical recognition of sEMG’s potential for predicting recovery is attributed to its provision of specific, quantitative data to inform treatment decisions (13). Therefore, the number of articles on the application of surface EMG in stroke has been increasing in recent years (14).

Bibliometric analysis, a quantitative method in the field of scientometrics (15), is extensively utilized for examining published literature across various domains, including stroke research (16, 17). This method can help quickly analyze the quantitative characteristics and laws of these literature, in order to provide direction and reference for future research. Employing bibliometric methods, this study analyzed the pertinent literature in the field of stroke research involving surface electromyography (sEMG). Since 2009, research on the sEMG assessment of stroke has expanded swiftly, with over 787 articles published, constituting approximately 86% of all related articles since 1979. Given this context, conducting bibliometric research on sEMG and stroke is imperative and could provide valuable insights for the field’s forthcoming developmental phase. Consequently, this study primarily employs the “bibliometrix” package (version 4.3.3) within the R environment to assess the current state of sEMG research post-stroke, offering a benchmark for subsequent research endeavors in this domain.

## 2 Materials and methods

### 2.1 Data sources and search strategies

**Figure 1:**
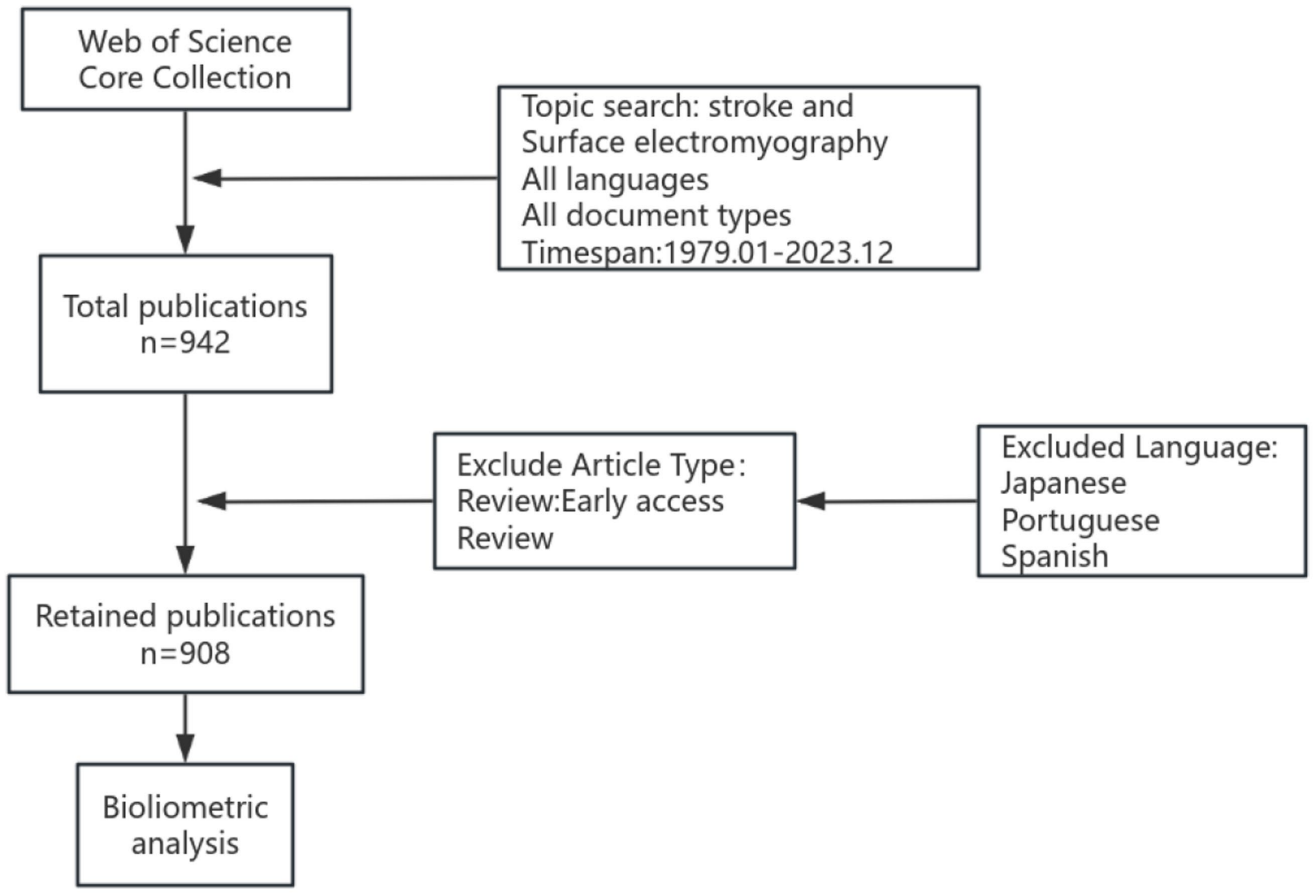
The flowchart of papers identification and selection.

Data were collected from the Web of Science Core Collection (WoSCC) database. WoSCC is an online platform facilitating the analysis of scientific research. WoSCC was chosen over other databases such as PubMed due to its comprehensive standardized datasets, detailed citation analysis tools, multidisciplinary coverage, and ability to provide a global research perspective, all of which are essential for robust bibliometric analysis (18, 19). Search strategy used in this study encompassed the terms of “stroke” and “surface electromyography,” and included various literature types such as article, article: early access, article proceedings paper, editorial material, letter, meeting abstract, and proceedings paper; reviews and review: early access were excluded. The search spanned papers from January 1, 1979, to December 31, 2023, resulting in 908 articles. The flowchart in Figure 1 depicts the process of papers identification and selection.

In R,^1^ the bibliographic information of the selected publications was automatically converted and analyzed using the “bibliometrix” package (20). We analyzed all information related to sources, countries, citations, world maps, institutions, authors, journals, and keywords.

## 3 Results

### 3.1 Data descriptive analysis

The results indicate that the mean citation rate for the 908 articles is 17.6%, including 7 document types: article, article: early access, article proceedings paper, editorial material, letter, meeting abstract, and proceedings paper. A total of 3,098 relevant authors were identified, with 21 being sole authors and 3,077 being multiple co-authors. The mean number of co-authors per paper was 4.81, and the proportion of internationally collaborating co-authors was 23.68%.

### 3.2 Global trends in the publications

A total of 908 articles related to stroke and sEMG, published between 1979 and 2023, were retrieved from the Web of Science Core Collection (WoSCC). The publication count rose from 1 in 1979 to 86 in 2023, illustrating a marked increase since 2009, at an average annual growth rate of 9.07%.

### 3.3 Analysis of citations

**Figure 2:**
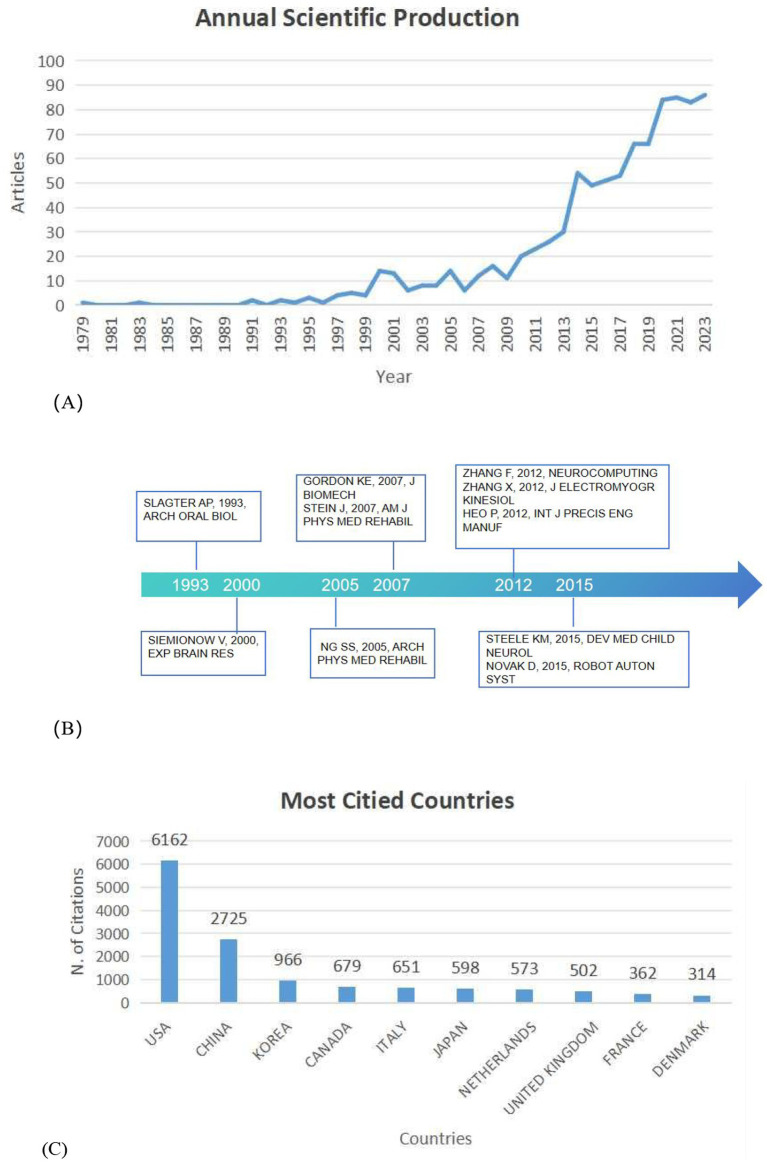
**(A)** The number of articles on stroke surface EMG per year. **(B)** Timeline of the top 10 cited documents. **(C)** Top 10 citied countries on sEMG in the field of stroke. The Top 10 citied countries refer to the countries of the corresponding authors of the publications in the field of surface electromyography (sEMG) for stroke rehabilitation. This information highlights the significant contributions these countries have made to the research in this field.

The analysis indicates that 18 articles have accumulated over 100 citations each. These articles span a range of topics, including 8 focused on clinical aspects, 3 centered on engineering fields, and an additional 7 that integrate both clinical and engineering perspectives. Figure 2B presents the top 10 most-cited documents, including an article with 429 citations titled “The timed up & go test: its reliability and association with lower-limb impairments and locomotor capacities in people with chronic stroke” (21). The study reported that the timed up & go test scores were reliable, were able to differentiate the patients from the healthy elderly subjects, and correlated well with plantarflexor strength, gait performance, and walking endurance in subjects with chronic stroke. Another highly cited article in Figure 2B with 343 citations titled “Current Hand Exoskeleton Technologies for Rehabilitation and Assistive Engineering” (22) offers an in-depth analysis of the most recent advancements in the domain of active hand exoskeletons, specifically examining their utility in rehabilitation and assistive robotics, the third sample from Figure 2B, titled “Learning to Walk with a Robotic Ankle Exoskeleton” (23) has 212 citations. This paper explored that robotic exoskeletons controlled by muscle activity could be useful tools for testing neural mechanisms of human locomotor adaptation. Additionally, Figure 2C indicates that the United States was the most frequently cited country, with 6,162 citations, followed by China with 2,725 citations.

### 3.4 Analysis of countries

**Figure 3:**
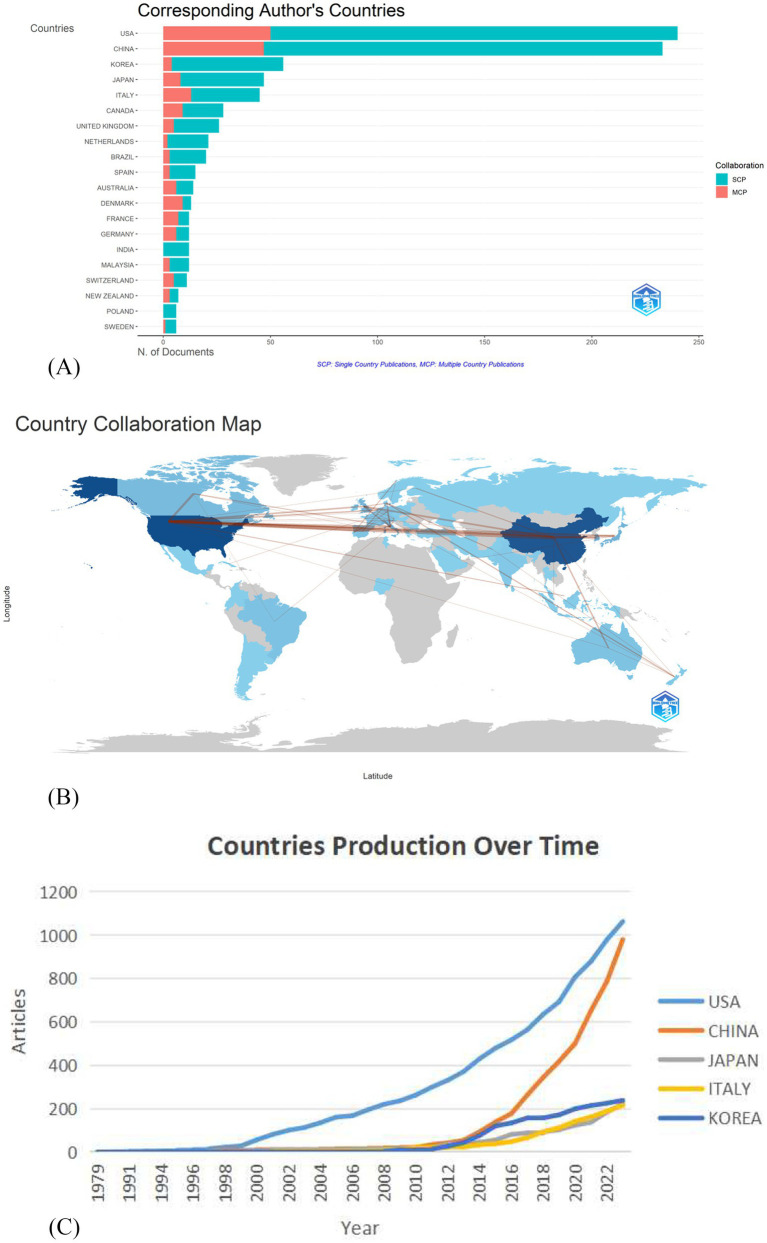
**(A)** Most productive countries and regions in the field of sEMG research on stroke. The red bars represent single Country Publications, which reflects a strong internal collaborative network in the subject area, facilitating the exchange of knowledge and innovation. The green bars indicate Multi-Country Publications, reflecting the country’s active role on the international stage and its ability to attract international cooperation, enhancing both the quality and international influence of scientific research. The figure illustrates the strong scientific research competitiveness of the United States and China. **(B)** The collaboration between countries and regions in sEMG research related to stroke is depicted. **(C)** The United States and China have published the most in recent years, with China’s growth rate being particularly noticeable.

Based on the countries of the corresponding authors, the top five countries with the most publications were the United States, China, South Korea, Japan, and Italy. The United States contributed the most articles (240, 26.4%), followed by China (233, 25.6%), South Korea (56, 6.1%), Japan (47, 5.1%), and Italy (45, 4.9%), as shown in Figure 3A. Furthermore, Figure 3B shows research collaboration among different countries/regions in stroke rehabilitation sEMG. Based on corresponding authors’ affiliations, it uses lines between countries to indicate cooperation strength. Colors on the map also matter: blue for countries with publications, grey for those without. In general, darker colors mean more published articles. Figure 3C illustrates the trends in country production over time in the field of surface electromyography (sEMG) for stroke rehabilitation. The graph shows the cumulative number of articles published by different countries from 1979 to 2023. The USA has consistently been a leading contributor, with a significant increase in publications since 2009, reflecting its strong research foundation and continuous investment in this area. China’s growth trajectory is particularly noteworthy, showing a substantial rise in publications especially after 2010, indicating its rapidly growing research capacity and interest in sEMG for stroke rehabilitation. Other countries like South Korea, Japan, and Italy also display gradual increases in publications, though at a slower pace compared to the USA and China. These trends highlight the increasing global attention toward sEMG in stroke rehabilitation, with the USA and China playing pivotal roles in driving the research forward.

### 3.5 Analysis of institutions

**Figure 4:**
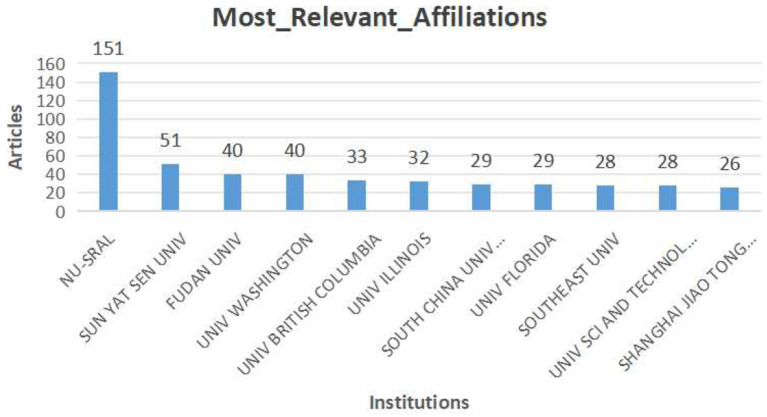
Top 10 institutions performing sEMG research works on stroke.

Figure 4 shows the institutions involved in the research, with a total of 1,278 institutions. Given the close collaboration between Northwestern University and Shirley Ryan AbilityLab, including shared researchers and numerous joint projects, their data have been combined. Together, they form the Northwestern University and Shirley Ryan AbilityLab Joint Research Consortium (NU-SRAL), which contributed 151 publications (16.6%). This is followed by Sun Yat-sen University (5.3%), Fudan University (4.4%), the University of Washington (4.4%), and University of British Columbia (3.6%).

### 3.6 Analysis of the authors

**Figure 5:**
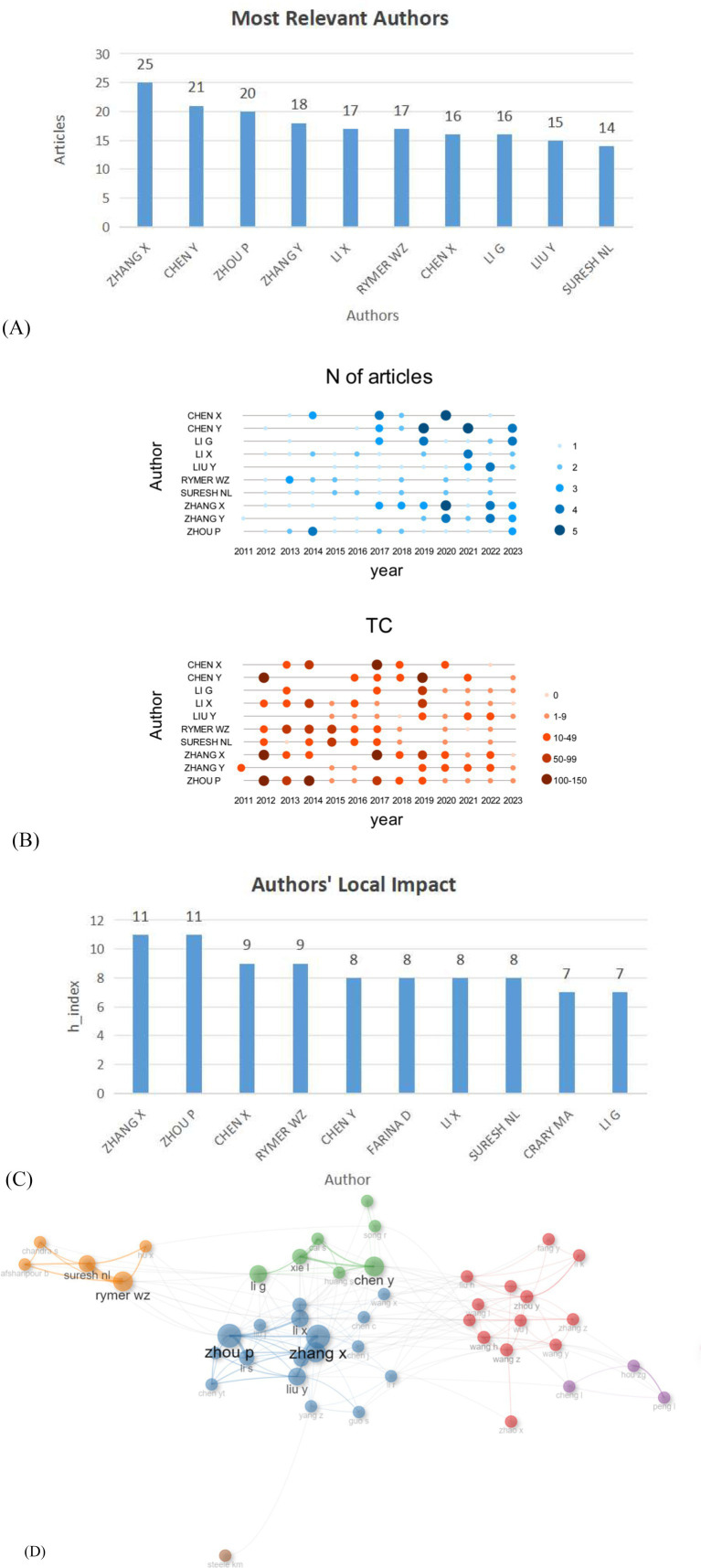
**(A)** Top 10 authors that produced sEMG research works on stroke. **(B)** Authors’ Production over Time. “N of articles” indicates the number of publications, while “TC” stands for “Total Citations,” representing the cumulative number of times the articles have been cited. The time of publication can be clearly and intuitively observed to understand the research hotspots in real time. **(C)** Authors’ Local Impact by H-index. H-index is an index used to measure the academic influence of scholars, scientists or researchers in bibliometrics. Specifically, if a scholar’s H-index is 10, it means that the scholar has at least 10 papers, each of which has been cited at least 10 times by other scholars. **(D)** The authors’ cooperative network on stroke surface electromyography. Each circle represents a different author, lines connecting the circles reflect connections between the authors, and variously colored networks of the linkages represent the groups of authors who frequently collaborate.

A summary of the top 10 most productive authors is presented in Figure 5A. Zhang X produced the most publications, with 25 articles (2.6%), followed by Chen Y with 21 articles (2.3%), Zhou P (20, 2.2%), Zhang Y (18, 1.9%), and Li X (17, 1.8%). As shown in Authors’ Production over Time in Figure 5B, each dot represents the number of articles published by an author in a specific year, with the size of the dot indicating the quantity. This visualization helps identify trends in research output and the periods of peak activity for each author, reflecting the evolving nature of research interest and focus over time. It can be observed that Zhang X has maintained a relatively consistent output of articles and a stable number of citations in recent years, whereas Zhou P, despite a relatively smaller output in recent years, has increased production since 2023. Furthermore, regarding the authors’ local impact as depicted in Figure 5C. A higher H-index is often associated with a greater influence and contribution to the research community, though it may not always be the sole indicator of a researcher’s impact and value. Zhang X and Zhou P have the highest impact, with an H-index of 11. Finally, the collaboration map in this field was analyzed for each author (Figure 5D), revealing that Zhang X and Zhou P have been involved in the most collaborations. Zhang X from the University of Science and Technology of China mainly focuses on surface electromyography (sEMG) signal processing, analysis, and application (24). His work covers signal denoising (25), muscle synergy analysis (26), deep - learning - based pattern recognition (27), cross - user gesture recognition (28), and clinical applications like cerebral palsy (29), stroke rehabilitation (30), and muscle strength estimation (31). Zhou P’s research focuses on the decomposition, processing, and application of surface electromyography (sEMG) signals. He has developed innovative high-density sEMG decomposition techniques, including a novel framework based on iterative convolution kernel compensation and an energy-specific peel-off strategy (32), as well as methods for online sEMG signal decomposition (33). His work also explores muscle neurophysiology, such as the relationship between motor unit characteristics and muscle fatigue (34), and uses single-fiber EMG to analyze post-stroke muscle changes (35). Furthermore, Zhou P has contributed to the development of high-density sEMG decomposition technology for motor unit number estimation (36) and employed three-dimensional innervation zone imaging technology (3DIZI) to assess muscle innervation zone distribution in stroke patients (37). However, it is important to note that there is a citation gap between engineering and clinical fields. Both Zhang X and Zhou P are engineers whose work has demonstrated certain clinical relevance, providing valuable insights for stroke rehabilitation and contributing to the development of more effective rehabilitation strategies.

### 3.7 Analysis of journals

**Figure 6:**
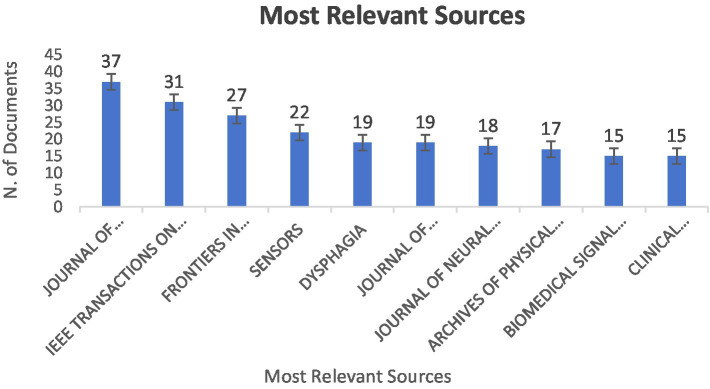
Top 10 journals publishing sEMG studies on stroke.

The 908 analyzed articles were published in 371 journals, Among the 371 journals, 73 are classified as clinical journals, accounting for approximately 19.68% of the total. Another 145 are classified as engineering journals, making up about 39.08% of all journals. The remaining 153 journals, which account for approximately 41.24%, are categorized as other types of journals. Figure 6 displays the top 10 journals with the largest number of publications in the field. The highest number of publications originated from the Journal of Electromyography and Kinesiology (37, 4.0%), followed by the IEEE Transactions on Neurological Systems and Rehabilitation Engineering (31, 3.4%), Frontiers in Neurology (27, 2.9%), Sensors (22, 2.4%), and Dysphagia (19, 2%). Of the top 10 journals, the IEEE Transactions on Neurological Systems and Rehabilitation Engineering (4.9) had the highest impact factor (IF). The journal is an academic publication by the IEEE (Institute of Electrical and Electronics Engineers), focusing on the fields of neural engineering and rehabilitation engineering.

The Journal of Electromyography and Kinesiology, the IEEE Transactions on Neurological Systems and Rehabilitation Engineering, and Sensors are predominantly engineering journals, while the Archives of Physical Medicine and Rehabilitation and Clinical Neurophysiology are more clinically oriented. The Archives of Physical Medicine and Rehabilitation centers on applying sEMG in rehabilitation practice, offering clinician’s vital guidance to enhance post - stroke motor function using this technology. Clinical Neurophysiology delves into sEMG’s role in assessing muscle and neural functions from a neurophysiological perspective, shedding light on post - stroke muscle changes and neural recovery mechanisms. This distinction highlights a notable gap between technical knowledge and its clinical relevance or application.

In the Journal of Electromyography and Kinesiology, many articles focus on the signal processing techniques of surface Electromyography (sEMG). These engineering-focused articles often have limited direct clinical application. On the other hand, journals such as Archives of Physical Medicine and Rehabilitation and Clinical Neurophysiology have emphasized the clinical application of sEMG in clinical assessment. A study evaluated the impact of the Ekso™ exoskeleton on gait in chronic stroke patients using sEMG and other technologies. It found increased prefrontal cortex activation during assisted walking and a link between non-affected limb muscle hypoactivation/coactivation and heightened prefrontal metabolism. This shows sEMG can offer key insights into the neural mechanisms of stroke patients’ gait disorders in clinical assessment (38).

In the field of stroke rehabilitation, most research on surface electromyography (sEMG) is published in engineering journals, but only a small portion directly talks about clinical applications. On the other hand, clinical journals publish a higher proportion of articles that focus on using sEMG in rehabilitation settings (39).

This discrepancy indicates a significant gap between sEMG technological advances and their clinical application. Innovative sEMG signal processing methods in engineering journals often lack clinical validation. Clinical journals, while reporting the effectiveness of sEMG - based assessment usually omit detailed technical descriptions needed for broad adoption. To address this, studies suggest integrating sEMG recording and analysis into physiotherapists’ education to reduce technical barriers and bridge theory and practice by simplifying sEMG and highlighting its clinical benefits (40). Furthermore, more interdisciplinary research combining engineers’ technical skills with rehabilitation professionals’ clinical insights is necessary. Such collaboration can yield sEMG technologies that are both innovative and effective in real - world rehabilitation settings.

### 3.8 Analysis of keywords

**Figure 7:**
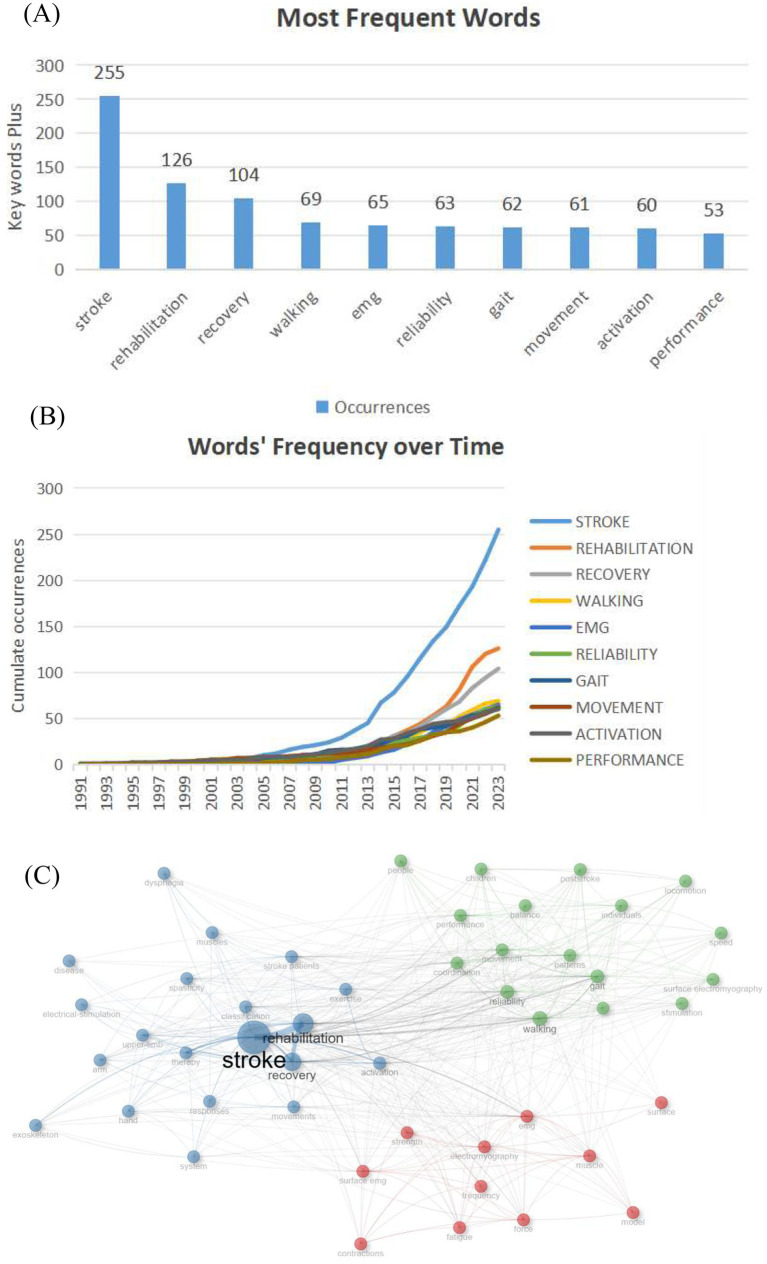
**(A)** Top 10 Frequent Words of sEMG studies on stroke. **(B)**The words frequency over time. **(C)** Co-occurrence Network Analysis of Stroke Rehabilitation Research.

We identified 238 keywords each mentioned over five times. Figure 7A lists the most relevant ones. “Stroke” was the most frequent (255 mentions, 12%), followed by “rehabilitation” (126, 6%), “recovery” (104, 5%), “walking” (69, 3%), “EMG” (65, 3%), and “reliability” (63, 3%). Figure 7B shows the top 10 keywords in recent sEMG stroke studies, with “stroke” being the most common. However, given “stroke” was a search keyword, its high frequency is expected and might not fully capture the field’s diverse research hotspots. The keyword “rehabilitation” reflects a trend toward comprehensive post - stroke rehabilitation interventions, covering various therapies such as physical, occupational, and speech therapy. It indicates a move toward multi - disciplinary approaches to enhance functional outcomes in stroke patients. For example, studies on task - specific training, robot - assisted therapy, and virtual - reality - based rehabilitation have shown great potential in improving motor function and activities of daily living (41). Notably, “EMG” ranked fifth with 65 mentions. Its presence underscores the technical foundation of sEMG in stroke rehabilitation research, indicating that many studies utilize EMG technology to assess muscle activity and motor function. This highlights the importance of EMG as a tool for planning and monitoring the effectiveness of rehabilitation interventions. Figure 7C presents a visual representation of the keyword co-occurrence network in stroke rehabilitation literature, where node size is proportional to the frequency of citation within the corpus. The most prominent nodes associated with the keywords “stroke,” “rehabilitation,” and “walking” indicate their prevalent use and pivotal role in shaping the research discourse. Monitoring the introduction of new keywords and the intensification of co-occurrence patterns can serve as a barometer for emerging research trajectories (42). The increasing visibility and clustering of terms like “AI-driven sEMG analysis” and “high-density sEMG” suggest these may be burgeoning areas of scholarly inquiry, pointing toward future research opportunities and potential growth sectors within the field (43, 44). These trends indicate that stroke rehabilitation research is evolving into a more holistic and technology-oriented domain, with a pronounced focus on assessing the impact of therapeutic interventions.

## 4 Discussion

### 4.1 The increasing importance of sEMG in stroke rehabilitation

Surface electromyography (sEMG) is a vital tool in stroke rehabilitation research, offering insights into muscle activation and motor function recovery. Since 2009, there has been a significant increase in sEMG - related research, indicating its potential to improve clinical practice and policy - making. This technology plays a crucial role in addressing the growing burden of stroke, especially in countries with aging populations like the US, China, South Korea, and Japan.

The value of sEMG in stroke rehabilitation lies in its effectiveness. It can assess muscle strength, coordination, and movement patterns, providing quantitative data for rehabilitation treatment. For instance, Zhang (45) found that the REX exoskeleton rehabilitation robot was more effective than traditional training in promoting early recovery in sub - acute stroke patients. Another study showed that a combined sEMG and accelerometer (ACC) wearable sensor system could automatically recognize motor tasks and assess functional independence in stroke patients (46). These studies highlight the diverse applications and potential of sEMG in assessing rehabilitation outcomes.

However, it is important to note that there is a citation gap between engineering and clinical fields. Engineering works are predominantly cited by other engineers rather than MDs. This suggests a disconnection between the engineering and clinical communities, potentially limiting the translation of engineering innovations into clinical practice. The clinical application of engineering innovations is indeed limited. Many engineering advancements, though technically remarkable, may not be widely adopted in clinical settings due to various barriers. These include the lack of STEM (Science, Technology, Engineering and Mathematics) education among clinicians, which may make them less confident in adopting a STEM approach to patient evaluation. Other barriers include lack of awareness, complexity of integration, the cost of sEMG equipment and testing not being covered by medical insurance, and insufficient evidence of clinical benefit (47).

### 4.2 Interdisciplinary collaboration and clinical relevance

A physiotherapist once said, “Engineering literature is offering us racing cars when we cannot even ride a bicycle.” This metaphor vividly reflects the current gap between engineering advancements and clinical application. While engineering research continues to push the boundaries of technology, offering sophisticated and innovative solutions, clinicians may find themselves unprepared to harness these advancements due to a lack of foundational technical training or resources. As noted earlier, this disconnection can limit the translation of engineering innovations into clinical practice, highlighting the importance of interdisciplinary collaboration and education to bridge this divide. To bridge this gap, it is crucial to enhance collaboration between engineers and clinicians. A successful example is the Center for Healthcare Engineering and Patient Safety (CHEPS) at the University of Michigan, established jointly by the College of Engineering and the Medical School (48). CHEPS aims to improve the safety and quality of healthcare delivery through a multidisciplinary approach based on systems engineering. The center focuses on developing innovative solutions to real-world healthcare problems while educating engineering students for careers in healthcare delivery. CHEPS strives to employ a “truly interdisciplinary approach,” where engineers and clinical partners engage in mutually beneficial learning, thereby increasing their understanding of each other’s domain strengths. Interdisciplinary collaboration is crucial for encouraging joint research projects and multidisciplinary teams—comprising both engineers and clinicians—to be involved in the design and implementation of large-scale clinical studies from the outset. Additionally, it is vital to develop mechanisms that translate engineering research into formats that are accessible and comprehensible to clinicians, such as clinical guidelines or user-friendly technologies. Moreover, educational programs combining engineering and clinical perspectives can train a new generation of professionals to work across both fields. This includes using suitable textbooks, tutorials and manuals prepared by experts on sEMG clinical use for educational applications (44).

By taking these steps, we can work toward a more integrated approach where engineering innovations are more readily adopted and applied in clinical practice, ultimately benefiting patient care.

### 4.3 Impact on clinical practice and policy

The integration of sEMG technology into stroke rehabilitation is significantly influencing clinical practice and policy. The Consensus for Experimental Design in Electromyography (CEDE) project provides valuable guidance on many issues including electrode selection for EMG studies (49), which is crucial for ensuring the validity and reliability of sEMG recordings in clinical settings. By following the recommendations outlined in the CEDE project, researchers and clinicians can make informed decisions about electrode types and location for specific applications. This ensures that the sEMG data collected is of high quality and can be accurately interpreted to inform clinical decisions.

Recent advancements in sEMG techniques have enabled more detailed analysis of muscle activity and movement patterns, helping in designing more effective rehabilitation interventions. These techniques have been successfully applied in various studies to evaluate the effectiveness of different rehabilitation approaches, such as robot-assisted therapy and EMG-driven neuromuscular electrical stimulation (50).

However, the translation of these technological advancements into routine clinical practice remains a challenge. There is a need for further research to establish clear guidelines on how to best incorporate sEMG into clinical workflows and to demonstrate its cost-effectiveness and impact on patient outcomes. Additionally, policy makers should consider supporting the integration of sEMG technology into rehabilitation services by providing adequate funding and resources for training and equipment. Gupta and Aggarwal (51) indicate that using sEMG - based and other metrological methods in rehabilitation demands intensive learning, so educational - policy upgrades are needed. This implies that variations in rehabilitation - science education policies and priorities among countries and institutions affect the clinical application and efficacy of sEMG technology.

### 4.4 Future directions and challenges

The field of sEMG in stroke rehabilitation continues to evolve rapidly, presenting both exciting opportunities and significant challenges. The standardization of sEMG methods remains a critical issue. The CEDE project and the JEK tutorials made substantial progress in this area by developing a series of matrices that guide decision-making in recording, data analysis, and interpretation of EMG studies. These resources provide researchers and clinicians with the tools needed to ensure the quality and validity of sEMG research and its application in clinical practice.

Another important consideration for the future is the integration of sEMG with emerging technologies such as artificial intelligence (AI). AI has the potential to enhance the analysis and interpretation of sEMG data, enabling more personalized and effective rehabilitation strategies. For instance, AI - powered gesture recognition, combined with wearable rehabilitation gloves based on sEMG systems, is being researched to boost hand rehabilitation (52). However, this also requires new educational and training programs for healthcare professionals to ensure they are equipped with the skills needed to leverage these technologies effectively. The complexity of AI-driven sEMG analysis may require the formation of interdisciplinary teams that combine the expertise of rehabilitation engineers, data scientists, and healthcare providers. This shift toward interdisciplinary collaboration in clinical settings will be crucial for maximizing the benefits of AI in sEMG applications.

In addition to AI, EMG-driven feedback and robot control applications are gaining increasing attention. Recent research indicates that EMG-driven feedback, by offering real-time information to patients and therapists, can effectively enhance motor learning and functional recovery, thereby improving rehabilitation outcomes (53). Moreover, the implementation of High Density sEMG (HDsEMG) is an emerging field that provides detailed muscle activity mapping and improved signal resolution (54). This advancement has the potential to significantly deepen our understanding of muscle function and rehabilitation strategies (55).

Furthermore, the increasing availability of sEMG technology and its potential applications in various clinical and research contexts highlight the need for continued education and training. As noted in recent publications, there is a growing recognition of the importance of equipping healthcare professionals with the knowledge and skills required to utilize sEMG effectively. This includes not only technical training in the use of sEMG equipment and software but also a deeper understanding of the underlying principles of electromyography and its clinical applications. For instance, the Consensus for experimental design in electromyography (CEDE) project: Checklist for reporting and critically appraising studies using EMG (CEDE-Check) provides a comprehensive framework for ensuring the quality of EMG research reporting and critical appraisal skills, which are essential for the education and training of healthcare professionals (49, 56–59).

In clinical practice, sEMG application faces practical barriers. For example, Goffredo (60) highlights technical challenges in sEMG assessment during robot-assisted gait training in acute stroke patients. This indicates a need to address these challenges in practical application. Additionally, Medved (61) critically evaluate sEMG as a teaching subject and clinical tool in medicine and kinesiology, emphasizing the need to integrate sEMG’s theoretical knowledge and practical skills. The application of machine learning in sEMG data analysis also offers new directions for future rehabilitation practice. For instance, Moniri et al. (62) used machine learning for real-time forecasting of sEMG features related to trunk muscle fatigue, while Zhong (63) employed fusion learning for sEMG recognition of multiple upper-limb rehabilitation movements. These studies provide strong support for improving rehabilitation outcomes and efficiency.

By taking these steps, we can work toward a more integrated approach where engineering innovations are more readily adopted and applied in clinical practice, ultimately benefiting patient care. This bibliometric analysis will hopefully help to achieve this goal.

## 5 Limitation

It is important to acknowledge the limitations of this bibliometric analysis, particularly the fact that not all articles and journals are included in the Web of Science Core Collection. This limitation extends to non-English language journals, which are often omitted from such databases. In addition, literature statistics from institutions may affect the results due to changes in their names. Finally, because of different algorithms while using R-based bibliometric tools to generate visual maps, there are no unitary setting processes of time division, threshold, and clipping methods, which may produce some deviation.

## 6 Conclusion

This study offers a comprehensive overview of research trends in surface electromyography (sEMG) for stroke rehabilitation. Since 2009, there has been a significant increase in studies highlighting the potential of sEMG in this field. The US and China are leading in research output and citation impact. Keyword analysis shows that “rehabilitation” and “recovery” are key research themes, reflecting a focus on improving post-stroke functional outcomes.

Despite the growing research interest, there remains a gap between engineering advancements and clinical application, indicating a need for better translation of engineering innovations into clinical practice. Future research should focus on the clinical utility of sEMG, including cost-effectiveness and integration into clinical workflows, while developing clear application guidelines. The standardization of sEMG methods and integration with emerging technologies like artificial intelligence present significant opportunities for advancing stroke rehabilitation. By addressing these challenges and fostering interdisciplinary collaboration, sEMG can play a crucial role in enhancing rehabilitation outcomes and improving the quality of life for stroke patients.

## Data Availability

The raw data supporting the conclusions of this article will be made available by the authors, without undue reservation.

## Footnotes

^1^R Project for Statistical Computing, http://www.r-project.org/ (accessed on March 20, 2024), version 4.3.3.
